# Bursite tuberculeuse de l’épaule chez une insuffisante rénale chronique: à propos d’un cas

**DOI:** 10.11604/pamj.2018.30.182.12557

**Published:** 2018-06-27

**Authors:** Mohamed Ben Jemaa, Wassim Zribi, Emna Elleuch, Wajdi Bouaziz, Ameur Abid, Soumaya Yaich, Mourad Aoui, Kamel Ayedi, Mounir Ben Jemaa, Mohamed Zribi, Hassib Keskes

**Affiliations:** 1 Service de Chirurgie Orthopédique et Traumatologique, CHU Habib Bourguiba de Sfax,Tunisie; 2 Service des Maladies Infectieuses, CHU Hédi Chaker de Sfax, Tunisie; 3 Service de Néphrologie, CHU Hédi Chaker de Sfax,Tunisie

**Keywords:** Bursite, épaule douloureuse, tuberculose, insuffisance rénale chronique, Bursitis, painful shoulder, tuberculosis, chronic renal failure

## Abstract

La tuberculose de l'épaule est rare. Elle regroupe toutes les atteintes tuberculeuses des tissus articulaires et péri-articulaires de l'épaule. Son évolution insidieuse mimant les pathologies inflammatoires et dégénératives, explique la fréquence de son retard diagnostique. Nous rapportons un cas rare d'une bursite tuberculeuse de l'épaule chez une femme de milieu rural, insuffisante rénale et traitée pour une tuberculose péritonéale et du psoas. Des signes d'imprégnation tuberculeuse ont été notés à l'anamnèse. L'examen clinique trouvait une tuméfaction douloureuse de l'épaule avec raideur. L'IRM de l'épaule était en faveur d'une bursite infectieuse. L'origine tuberculeuse était confirmée par l'examen histologique d'une biopsie synoviale écho-guidée. Un traitement antituberculeux lui a été instauré avec bonne évolution. Au recul de 9 ans, elle retrouve une fonction articulaire satisfaisante avec absence de récidive infectieuse.

## Abstract (en)

Tuberculosis of the shoulder is rare. It encompasses all articular and periarticular tuberculouses of the shoulder. Its insidious evolution, mimicking inflammatory and degenerative diseases, reflects the frequency of its diagnostic delay. We report a rare case of tuberculous bursitis of the shoulder in a woman living in rural areas, with renal insufficiency and treated for peritoneal TB and psoas. The anamnesis revealed signs of tuberculous impregnation. Clinical examination showed painful swelling of the shoulder associated with stiffness. MRI of the shoulder objectified infectious bursal disease. Its tuberculous origin was confirmed by the histological examination of ultrasound-guided synovial biopsy. The patient underwent TB treatment with good outcome. At 9-year follow-up, the patient had satisfactory articular function with no recurrence of infectious disease.

## Introduction

La bursite tuberculeuse de l'épaule est une forme rare des tuberculoses de l'épaule. Son tableau clinique, caractérisée par une douleur et une raideur articulaire chronique, peut errer le diagnostic vers une atteinte dégénérative ou inflammatoire expliquant ainsi le retard diagnostique et thérapeutique. A travers un cas d'une bursite tuberculeuse chez une femme insuffisante rénale chronique et aux antécédents de tuberculose traitée, nous essayons de dégager les caractéristiques cliniques et paracliniques de cette entité pathologique et nous mettons au point les particularités de sa prise en charge thérapeutique.

## Patient et observation

**Figure 1:**
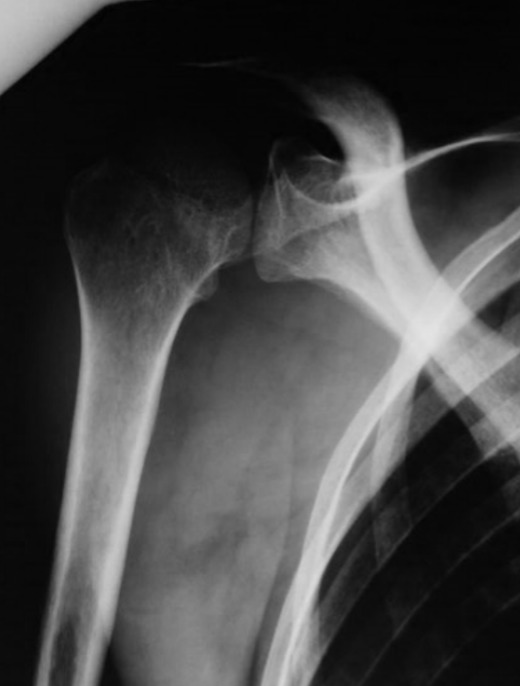
Radiographie standard de l’épaule (incidence de face), une déminéralisation osseuse péri-articulaire

**Figure 2:**
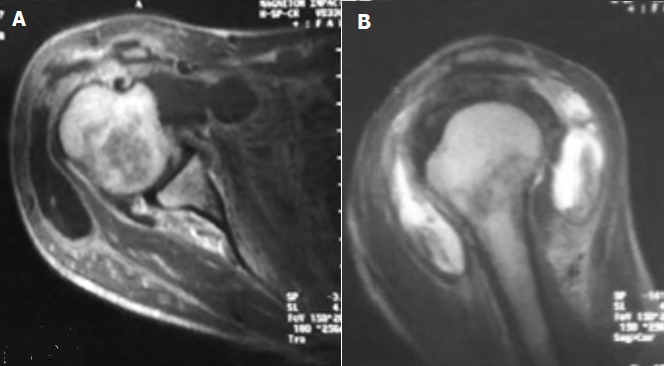
IRM de l’épaule (A) coupe axiale; (B) coupe frontale), distension de la bourse synoviale sous acromio-deltoïdienne par un épanchement liquidien de grande abondance avec épaississement synovia

Il s'agit d'une femme de 55 ans issue d'un milieu rural, aux antécédents d'une insuffisance rénale chronique au stade d'hémodialyse, une tuberculose péritonéale et un abcès tuberculeux du psoas traités. Elle se plaignait de douleurs chroniques de l'épaule d'évolution insidieuse non améliorées par le traitement symptomatique avec apparition récente d'une tuméfaction en regard d'aggravation progressive. L'anamnèse rapportait une altération récente de l'état général avec fièvre, asthénie, anorexie et amaigrissement et des sueurs nocturnes. L'examen clinique trouvait une patiente amaigrie et fébrile. L'épaule était enraidie, tuméfiée et de mobilisation douloureuse. Une rougeur et une chaleur locale étaient trouvées. Le reste de l'examen ostéo-articulaire était sans particularités en particulier celui du rachis cervical. L'IDR à la tuberculine était négative. Le bilan biologique a objectivé un syndrome inflammatoire (CRP à 45mg/l et VS de 105mm à la première heure). La leucocytose était normale (Globules blancs à 6600/mm^3^). La recherche de bacilles de Koch (RBK) dans les crachats et les urines était négative. La radiographie standard de l'épaule a montré une déminéralisation osseuse locorégionale (Figure 1). L'échographie a montré un épanchement de grande abondance de la bourse sous acromio-deltoïdienne. Elle était complété par une ponction biopsie écho-guidée. Une exploration par IRM a été pratiquée. Elle a montré une bursite sous-acromio deltoïdienne avec un épaississement synovial et un épanchement liquidien de grande abondance hétérogène étendu aux faces antérieures et latérales de l'épaule et un épanchement minime de l'articulation gléno-humérale. Par contre aucune atteinte osseuse n'a été trouvée (Figure 2).

L'examen bactériologique du liquide de ponction trouvait un liquide trouble dont l'examen direct et la culture dans les milieux standard ainsi que la RBK étaient négatifs. L'examen anatomopathologique de la biopsie écho-guidée a montré une inflammation synoviale spécifique contenant des granulomes tuberculoïdes centrée par une nécrose caséeuse confirmant ainsi l'origine tuberculeuse. Un traitement antituberculeux lui a été instauré. La durée totale du traitement était de 12 mois. Le schéma du traitement était comme suit: quadrithérapie initiale pendant 2 mois associant les antituberculeux suivants; rifampicine: 10 mg/kg/ 24 heures; isoniazide: 5 mg/kg/ 48 heures; pyrazinamide: 30 mg/kg/ 48 heures; ethambutol: 20 mg/kg/48 heures. L'isoniazide, le pyrazinamide et l'ethambutol étaient administrés après la séance d'hémodialyse. Relais par une bithérapie antituberculeuse à base de rifampicine et isoniazide prescrit aux mêmes posologies pendant 10 mois. L'observance et la tolérance thérapeutique étaient bonnes. L'évolution était favorable avec sédation progressive des douleurs et des signes inflammatoires locaux et une disparition du syndrome inflammatoire biologique. Une raideur modérée était bien jugulée par une rééducation de l'épaule commencée au cours du traitement et après accalmie des douleurs. Au recul de 9 ans, la patiente retrouve une fonction quasi-normale du membre avec récupération des amplitudes articulaires et absence de récidive infectieuse.

## Discussion

La tuberculose de l'épaule représente 1 à 2% des tuberculoses ostéo-articulaires [1]. Elle englobe l'atteinte par Mycobacterium tuberculosis non seulement des structures articulaires mais encore péri-articulaires de l'épaule. La bourse sous acromio-deltoïdienne représente une localisation exceptionnelle. E.Pertuiset considère que les bursites tuberculeuses font partie des tuberculoses ostéo-articulaires. Elles sont dues à un ensemencement d'origine hématogène de la synoviale de la bourse séreuse [2]. L'inoculation directe et la contamination par contiguïté ont été décrites [3]. Il existe deux formes anatomopathologiques des bursites tuberculeuses: la forme exsudative avec distension de la bourse séreuse et la forme abcédée avec petits abcès et des calcifications. Une atteinte des parties molles et des structures ostéo-articulaires adjacentes par contiguïté peut se voir à un stade avancé de leur évolution [2]. Comme toute tuberculose périphérique, l'atteinte de l'épaule se voit préférentiellement chez des terrains d'immunodépression particuliers tels que: l'insuffisance rénale chronique notamment hémodialysé, la transplantation rénale, le diabète, une pathologie tumorale maligne, l'éthylisme, la cirrhose éthylique, les connectivites, la corticothérapie au long cours et les traitements immunosuppresseurs [2,4, 5]. Selon E.Pertuiset et C.Ben Taarit, l'existence des antécédents d'une tuberculose constitue le principal facteur favorisant la survenue d'une tuberculose périphérique [2, 4]. Le tableau clinique des tuberculoses de l'épaule est caractérisée par une douleur chronique enraidissante pouvant égarer le diagnostic vers une atteinte dégénérative ce qui explique le retard diagnostique et les dégâts ostéo-articulaires parfois constatées [1, 6, 7]. Dans une série chinoise de 16 cas de TBC de l'épaule, J.Q Li rapporte 14 cas dont le diagnostic a été porté initialement pour une l'épaule bloquée [7]. La fistule cutanée et l'abcès froid sont caractéristiques mais rares [7].

La limitation de la mobilité articulaire peut se traduire à long terme par une amyotrophie du muscle deltoïde [7]. Une variété fulminante aiguë simulant une arthrite septique ou ostéosarcome, a été décrite par J.Mangwani [1]. La radiographie standard peut monter en cas d'une bursite tuberculeuse, des signes osseux en regard de la bourse atteinte à type d'une ostéopénie accrue, un amincissement cortical localisé, des érosions osseuses, un épaississement des parties molles avec des calcifications, une ostéolyse et des ostéo-condensations à un stade plus tardif [2, 8]. Le tableau radiologique des tuberculoses de l'épaule est caractérisé par les lésions souvent destructrices. Dans une série turque de 11 cas de tuberculose de l'épaule, 8 cas (72.8%) étaient aux stades radiologiques III et IV de la classification de M. Martini [9]. L'échographie montre un épaississement synovial avec un épanchement de la bourse. Cet épanchement peut être échogène en rapport avec des calcifications de petites tailles appelées des grains de « riz » [8]. Elle permet entre autre, guider les prélèvements bactériologiques et anatomopathologiques évitant ainsi le recours à la chirurgie. L'IRM est l'examen de choix permettant une meilleure analyse lésionnelle d'une tuberculose articulaire. Elle fait distinguer en cas d'une bursite deux formes: La forme exsudative avec distension de la bourse par un épanchement homogène et La forme abcédée contenant multiples abcès de petite taille avec des zones en hypo-signal T2 correspondant à la nécrose. La paroi de la bourse se rehausse après injection de Gadolinium et peut contenir des calcifications. L'aspect hétérogène est expliqué par la présence du matériel caséeux et de débris synoviaux. Les « grains de riz » sont mieux observés à l'RM sous forme de corps étrangers hypo-intenses [2, 8].

L'insuffisance rénale chronique constitue un terrain particulier nécessitant une preuve diagnostique certaine avant de démarrer un traitement antituberculeux prolongé. La certitude diagnostique peut être soit bactériologique par l'identification de Mycobacterium tuberculosis, soit anatomopathologique par la mise en évidence d'un granulome tuberculeux ou bien par l'isolement de l'ADN mycobactérien par PCR [9]. L'IDR à la tuberculine peut être négative en cas d'insuffisance rénale chronique, dénutrition, vieillissement, coïnfection par le VIH et les états d'immunodépression [2]. Le traitement des tuberculoses de l'épaule est essentiellement médical. De bons résultats fonctionnels étaient obtenu avec un traitement conservateur et une rééducation malgré une destruction articulaire avancée car il s'agit d'une articulation non portante du corps et supporte plus les irrégularités articulaires [1, 6, 9, 10]. La durée du traitement antituberculeux est de 12 mois prolongée si besoin [6]. Le recours à la chirurgie devrait être en dernier ressort en cas d'une mauvaise réponse au traitement médical ou en présence d'une destruction articulaire avancée. Elle consiste en une synovectomie et excision des tissus infectés [1, 6, 9]. Ogawa K. et ses collègues ont utilisé un système d'irrigation continue fermée associée à une aspiration douce à pression négative pour traiter 2 cas opérés de tuberculose de l'épaule. Ce système permet de faciliter l´élimination du tissu caséeux résiduel et rendant possible l'administration locale d'antituberculeux [6]. L'immobilisation de l'épaule au début du traitement doit se faire en abduction et rotation externe pour prévenir l'enraidissement en adduction et à visée antalgique. Cette immobilisation doit être de courte durée pour lutter contre l'ankylose articulaire [1]. La rééducation permet la restauration de la mobilité articulaire favorisant ainsi la réinsertion socio-professionnelle. Cependant elle doit être commencée dès la sédation des douleurs [2].

## Conclusion

La bursite tuberculeuse de l'épaule est rare. Il faut l'évoquer de principe devant toute douleur de l'épaule non améliorée par un traitement symptomatique surtout en milieu endémique et en présence de facteurs favorisants. Avoir une preuve diagnostique formelle d'une origine tuberculeuse est un enjeu important chez un insuffisant rénale chronique avant de démarrer le traitement antituberculeux. Le traitement est essentiellement conservateur. La chirurgie garde quelques indications.

## Conflits d’intérêts

Les auteurs ne déclarent aucun conflit d'intérêts.
